# Listening Effort and Its Relation to Spatial Localization, and Vestibular and Visual Impairment in Usher Syndrome—Our Experience

**DOI:** 10.3390/audiolres15060169

**Published:** 2025-12-05

**Authors:** Tiziana Di Cesare, Paola Michieletto, Maria Teresa Bonati, Federica De Caro, Pietro Cossu, Francesco Torelli, Eva Orzan

**Affiliations:** 1 Audiology and ENT, Institute for Maternal and Child Health—IRCCS “Burlo Garofolo”, 34137 Trieste, Italy; federica.decaro@burlo.trieste.it (F.D.C.); pietro.cossu@burlo.trieste.it (P.C.); eva.orzan@burlo.trieste.it (E.O.); 2 Ophthalmology, Institute for Maternal and Child Health—IRCCS “Burlo Garofolo”, 34137 Trieste, Italy; paola.michieletto@burlo.trieste.it; 3 Clinical Epidemiology and Health Services Research, Institute for Maternal and Child Health—IRCCS “Burlo Garofolo”, 34137 Trieste, Italy; mariateresa.bonati@burlo.trieste.it; 4 Medicine, Surgery and Health Sciences, University of Trieste, 34137 Trieste, Italy; ftorelli99@gmail.com

**Keywords:** Usher syndrome, listening effort, localization, hearing loss, vestibular function, visual function

## Abstract

Background/Objectives: Children with hearing loss (HL) could experience significant fatigue which compromises their performance. The effort related to the combination of HL and visual impairment in children affected by Usher syndrome (USH) could compromise mental health, socio-emotional behavior and academic achievement. The aim of the present study was to analyse the listening effort in USH cases types 1 and 2 and its relation to age, molecular diagnosis, visual field, visual acuity, degree of HL, vestibular impairment and spatial orientation. Methods: This was a retrospective monocentric study. Twenty children with genetically confirmed USH (USH2 in 15/20–75% and USH1 in 5/20–25%), age range 3–17 years (mean 9.6 ± 4.7), underwent: the Vanderbilt fatigue scale questionnaire (VFS), audiological and vestibular assessment including the Oldenburg Matrix test in Italian and video head impulse test (VHIT), sound localization test and ophthalmologic examination. Results: We observed a more pronounced HL and deteriorated vestibular function in those with USH1. They also employed significantly more time and head movements to localize sounds compared to USH2 and had the worst visual field on eye examination. The VFS did not show significant differences between the two groups, with the exception of the physical fatigue reported by parents. Mean VFS was linearly related to age, the hearing threshold of the worse ear, data logging hours of hearing device, time and head movements of the localization test, VHIT asymmetry and balance problems referred by parents and the visual field. USH type 1 had no greater risk of fatigue than USH2. Profound hearing loss, data logging of hearing device < 8 h a day, difficult localization test, balance problems and low retinal sensitivity represented risk factors for listening effort measured with VFS. Conclusions: Listening effort in difficult environments such as school rooms in USH patients is not only associated to hearing function but also to the spatial awareness determined in part by vestibular and visual function. Teachers should be informed and made aware of multiple comorbidities in order to facilitate learning.

## 1. Introduction

Congenital sensorineural hearing loss (SNHL) is one of the most prevalent chronic conditions in children, affecting from 1 to 3 per 1000 newborns worldwide [1].

In children of primary school age, the prevalence increases, with a further increase during adolescence because of progressive, acquired or late-onset causes [2].

The improvement in universal newborn hearing screening programs made it possible to detect HL in the first days after birth in order to start hearing rehabilitation early with hearing aids or cochlear implants and allow adequate language development [3].

Advances in congenital cytomegalovirus testing, imaging modalities (CT and MRI) and genetic testing (e.g., whole exome sequencing, WES) have significantly changed the challenge of traditional clinical evaluations arriving at an etiologic diagnosis, providing clinical and prognostic data that can guide personalized hearing rehabilitation interventions. Despite this, the lack of resources, especially in disadvantaged geographical areas, often complicates the possibility of accessing optimal clinical care and hinders etiological identification, causing them to be classified as idiopathic cases [4]. Nevertheless, the definition of genetic causes (syndromic or not), which account for the majority of cases, helps predict possible threshold progression and identify associated conditions which assist in establishing a therapeutic alliance with the family as the child grows.

Usher syndrome (USH) is an autosomal recessive disorder characterized by hearing and visual (and sometimes vestibular) impairment due to pathogenic variants affecting protein complexes of the sensory hair cells in the inner ear and retina involved in the signal transduction or in the cell adhesion [5]. It is the most common cause of deaf/blindness, responsible for 50% of cases under the age of 65 (affecting approximately 1/10,000~400,000 people worldwide) [6], and it is clinically and genetically heterogeneous. The different phenotypic traits of USH can be classified into three different types: in USH1, congenital HL (often severe to profound) is associated with early onset of retinitis pigmentosa (RP) and vestibular deficit. USH2 is characterized by moderate to severe HL, later onset of RP and non-vestibular dysfunction, while USH3 has variable visual and vestibular impairments combined with progressive HL [7]. USH provokes great disability and limits social activities and spatial orientation.

Many studies have shown that children with HL experience significant fatigue which could compromise the child’s performance in the classroom [8]. In fact, acoustic degradation elicits perceptual uncertainty and the necessity of additional mental effort especially in reverberating and noisy backgrounds [9]. It has been demonstrated that the cognitive effort related to challenging listening situations interferes with resources required for memory load and selective attention capacities [10]. Multiple subjective (rating scale, questionnaires) and objective (pupillometry, skin conductance, electroencephalogram, functional near-infrared spectroscopy) measures of listening effort have been proposed to monitor and compare patients in order to adopt the best rehabilitation strategy [11]. The pediatric Vanderbilt fatigue scale (VFS) [12] is a sensitive, reliable and valid measure of listening-related fatigue in children that may be used in clinical practice to identify overloaded children and to examinate the effectiveness of potential interventions.

In syndromic patients the situation is more complicated since HL is associated with multiple disabilities. In fact, it has been demonstrated that auditory perception, especially in difficult environments, is facilitated by the integrity of the visual pathways and spatial perception. When one sense (e.g., audition) is not perfectly efficient, another sensory modality (e.g., vision) can be consequently strengthened in order to complete a certain task, on the basis of an innate process which can be increased through specific training programs, called “sensory-substitution” [13]. This is why the backward spatial perception (a region where humans are naturally blind) is possible via other sensory modalities (especially audition) [14]. The combined HL, visual impairment and sometimes balance problems in USH children could penalize the efficiency of this integrated sensory system, and the related increased effort compromises their mental health, socio-emotional behavior and academic achievement. Furthermore, since the phenotype and the gravity of clinical traits are variable but usually get worse with age, daily communication tasks become more and more cognitive and energy consuming, restricted to near surroundings with limited access to distant information and in need of the help of the few sensory modalities not affected by the disease (e.g., tact, smell). Consequently, early genetic confirmation is of primary importance so that the correct rehabilitation can start and the mode of communication customized, preventing possible psychological comorbidities [15].

The main aim of the present retrospective study was to measure the listening effort in our USH pediatric cases types 1 and 2, comparing the two groups of patients on the basis of genetic diagnosis.

Secondarily, considering the necessary integration of auditory information with spatial perception and therefore the integrity of the vestibular and visual pathways, especially in difficult environments, we tried to quantify any relationship between the subjective reported listening effort and age of patients, visual field, degree of HL, vestibular impairment and spatial orientation.

## 2. Materials and Methods

### 2.1. Study Sample

We performed a monocentric retrospective study approved by the Ethical Committee of Institute for Maternal and Child Health—IRCCS, “Burlo Garofolo”, Trieste, Italy, under the name and number “Ricerca Corrente 17/23”. We included children (<18 years) in follow-up at our Audiology and ENT Service affected by Usher syndrome who have received molecular genetic confirmation through NGS sequencing, either by a panel or whole exome approach.

Genetic tests were primarily performed at the Medical Genetics Laboratory of IRCCS Burlo Garofolo and the Medical Genetics Laboratory of the University Hospital of Padova.

We excluded patients with a follow-up period < 2 years after the molecular diagnosis. Attention deficit, verbal working memory or cognitive impairment represented exclusion criteria. Only children with an IQ > 85 were included in the study.

Parents received complete and comprehensible information about the tests administered and gave their written consent to their execution, in agreement with the ethical standards of the Declaration of Helsinki.

### 2.2. Design

#### 2.2.1. Each Patient Enrolled Underwent

A Vanderbilt fatigue scale (VFS) questionnaire for children (the version translated in Italian) [12]: a tool that evaluates the degree of listening-associated fatigue, one for the parent and one for the child. The VFS-P (Parent) is divided into two sets of questions, 7 questions for mental fatigue and 5 questions for physical fatigue. Each question receives a score between 0 and 4, with the total scores ranging between 0 and 28 for mental fatigue and 0 and 20 for the physical part. A score ≤ 14 in the mental section or ≤12 in the physical one indicates that fatigue-related issues are relatively infrequent, which can be found in normally developing children. A mental and physical score, respectively, between 15 and 24 or 13 and 17 indicates that listening-related fatigue is relatively common for the child and may deserve attention. A score between 25 and 28 or 18 and 20, respectively, indicates that fatigue is very common and can severely impact the child’s life. It was administered for parents of children > 3 years old.

The VFS-C (Child) is designed to assess listening-related fatigue in children between 6 and 17 years of age. It is composed of 10 questions with a score of 0–4, for a total score ranging from 0 to 40. A score of 0–26 indicates that listening-related fatigue is infrequent. A final score of 27–36 indicates that fatigue is relatively common, while a score of 37–40 that listening-related fatigue is very common and impacts the child’s life. It was administered in children ≥ 6 years old.

Tonal Audiometry and Speech discrimination in quiet: We measured pure tone average (PTA) (average of hearing threshold levels at 500, 1000, 2000, and 4000 Hz) for both sides with and without hearing devices (hearing aids/cochlear implants). Speech discrimination of disyllabic words in quiet was also calculated considering the percentage of words the patient could recognize at 50 dB, 65 dB and 80 dB with their hearing devices. We considered the worse ear to compare patients.

Oldenburg Matrix test in Italian: Speech intelligibility in noise was evaluated with the use of the Sentence Matrix test in Italian [16] in children ≥ 6 years old. It is a test in which the patient is asked to repeat sentences of 5 words that are presented together with a disturbing noise (more correctly called “competition”). After each patient response, the software automatically makes the next sentence “easier” or “harder” to understand, based on that response, increasing the intensity of noise. The patient sits one meter in front of a speaker that sends the signal at a fixed intensity of 65 dB HL. We performed the test sending signals and noise from the same loudspeaker in front of the patient (0–S0N0). The test converges to a ratio of speech versus noise level in which the patient understands about 50% of the words presented. The result of this test is a value, called the Signal Reception Threshold (SRT), which represents the difference between the volume of the words and that of the noise at which the patient guesses half of the words presented. Consequently, lower numbers indicate better sentence recognition performance. The average value for the Italian population is −7.3 + 0.2 dB [16]. However, patients with hearing loss may have higher SRTs, even above zero. We used the version for children in case of age < 10 years, otherwise the version for adults.

Localization test: This test took place in a room that contains several rings of speakers at different heights [17] for children ≥ 6 years old. The patients were asked to sit in the middle of the room while wearing VR goggles (Oculus quest 2) and to hold a joystick in each of their hands. The test included 48 trials, during which a pulsating pink noise was played by six speakers at 15°, 45° and 75° on both the right and the left of the individual. Each patient had to point the joysticks toward where they heard the sound coming from. The purpose of the test was to determine their ability to correctly locate where a sound is coming from (sound spatial orientation), calculating: (1) their absolute mean error (in angular degrees—°) in localizing the sound with standard deviation; (2) how many seconds (s) it would take to complete the test; and (3) how much (in meters—m) they would move their heads in the act of looking for the sound.

Vestibular examination: For each patient, we considered:

- The age they first started walking in months.
- A questionnaire to verify the presence of balance and instability issues (balance questionnaire—BQ) developed by the authors of this study. The survey included 10 questions, each one investigating the presence of balance problems in 10 different areas: running; riding a bike; roller skating; walking in the dark; walking on uneven surfaces; walking on sand; carsickness; gymnastics and physical activity; turning their head right and left as they ride a bike; and keeping balance under the shower. Each question should be answered with “yes” (1 point), “no” (0 point) or “don’t know” (0 point), obtaining a total score from 0 to 10. It was administered to parents.
- Clinical examination to assess the presence of spontaneous and/or positional nystagmus, positivity of head-shaking test (HST) and clinical head impulse test (HIT).
- Video Head Impulse Test (VHIT) [18] using a VOG device (ICS Impulse, GN Otometrics, Taastrup, Denmark), able to measure the gain of VOR (Vestibular-Oculomotor Reflex) in both sides. We evaluated only the horizontal canals with the patient sitting upright and fixating on a visual target in front of him. Clinicians standing behind the patient generated head impulses by moving it abruptly and unpredictably in the horizontal plane. VOR gain was automatically calculated by the system as the ratio of head to eye velocity. We considered normal as: VOR gain >0.8 for each side and gain asymmetry between the two sides < 20%.

#### 2.2.2. Ophthalmologic Examination

- Best corrected visual acuity (BCVA).
- Automated static perimetry (ZEISS, Humphrey Visual Field HFA 750, Oberkochen, Germany) with SITA Fast 30-2 protocol used to investigate the visual field sensitivity in decibel (dB) investigating retinal sensitivity presenting light spots of different luminance. We calculated the mean of the four quadrants (nasal inferior, nasal superior, temporal inferior, temporal superior) for each eye. We considered the worse eye to compare patients.
- Tomographic retinal evaluation using Optical Coherence Tomography (OCT Optovue AngioVue, Visionix, USAt in cooperative patients, and OCT Optovue iVue 80, for evaluations under sedation). The foveal margin can be typically seen as a ring-like reflection of the internal limiting membrane that measures around 1500 μm in diameter and a normal thickness of about 239 μm [19]. The parafovea is a belt that surrounds the foveal margin and measures around 0.5 mm in thickness, while the perifovea surrounds the parafovea and is 1.5 mm wide.
- Finally, we used fundus photography and autofluorescence to highlight alterations in the pigmented epithelium and accumulations or dispersions of lipofuscin. Fundus photography uses a series of mirrors and lenses to focus a donut-shaped light beam that enters the eye through the cornea, taking a picture of it. Fundus autofluorescence is a non-invasive exam that does not require the administration of exogenous dyes, but instead relies on the presence of naturally fluorescent substances in the retina, such as lipofuscin, to create an image. Abnormalities in FAF are defined as any pattern that differs from the classic appearance [20].

### 2.3. Statistical Analysis

Statistical analysis was performed using Excel for Windows software. The sample was described in its clinical and demographic features using descriptive statistics techniques. Continuous values with a normal distribution, such as the scores obtained with questionnaires, were expressed as mean ± standard deviation (SD). Qualitative variables were summarized with absolute and percentage frequency tables.

The study’s main objective was achieved by calculating for each patient the mean scores of VFS in the total sample and in the two different groups (USH1 and USH2). To determine significant differences between the two groups in the screened demographic and audiological continuous variables, due to the small number of subjects, we used nonparametric statistics for group comparisons (ANOVA test). The results were considered significant for *p* values < 0.05.

To calculate the relation between the listening effort and the other factors, we used the mean score (VSF-M) between the VFS-C and VFS-P. Multiple Pearson’s correlation analyses were performed and Bonferroni’s correction for multiple comparisons was applied to investigate the relationship between listening effort and eight different possible variables: age, hearing threshold, data logging of hearing device, visual field, localization test (time and head movements), VHIT asymmetry and balance questionnaire. We considered the *p* value < 0.006 (*p* 0.05/8 = 0.006) significant.

We also used the nonparametric chi-square test to assess potential risk factors for listening effort in USH patients. We considered a mental and physical score of, respectively, ≥15 and ≥13 for VFS-P and a score ≥ 27 for VFS-C. The results were considered significant for *p* values < 0.05.

## 3. Results

### 3.1. Patients

Twenty children with genetically confirmed Usher Syndrome (USH) (Table 1) were included in our study—age range 3–17 Years (Mean 9.6 ± 4.7)–10/20 (50%) M and 10/20 (50%) F–19/20 (95%) caucasian and 1/20 (5%) asian (N16, Table 2).

Genetic testing confirmed a diagnosis of USH2 in 15/20 (75%) and USH1 in 5/20 (25%). We reviewed the DNA variants identified in USH patients with the support of online databases, including ClinVar (https://www.ncbi.nlm.nih.gov/clinvar/, accessed on 2 January 2025), and HGMD^®^ Professional (https://digitalinsights.qiagen.com/) (accessed on 2 January 2025). Deeper details about molecular genetic diagnoses are reported in Table 1.

### 3.2. Audiological Assessment

The mean age for hearing loss (HL) diagnosis was 11.4 ± 20.3 months. It was statistically different (*p* = 0.024) between USH1 (mean 0 months, first identification with the neonatal hearing screening in all cases) and USH2 (15.2 ± 22.3 months) because of six cases with diagnostic delay (mean 38 months), three of them with a negative hearing screening test. The genetic diagnosis was confirmed at 6.9 ± 4.5 years, without difference between the two groups (*p* > 0.05).

The hearing threshold of the worst ear at the diagnosis and the last follow-up appointment was higher in USH1 (101 dB HL and 109 dB HL, respectively) compared to USH2 (56.9 dB HL and 60.6 dB HL, *p* = 0.015) without a significative progression in both groups. In any case, three USH2 patients had a severe to profound HL at the diagnosis.

In total, 19/20–95% patients used hearing devices (7/19–36.8% bilateral cochlear implants, 100% of USH1 and 13% of USH2, and 12/19–63.2% bilateral hearing aids, 0% of USH1 and 87% of USH2).

The mean age of first HA use was 30 ± 23 months and it was statistically different (*p* < 0.001) between the two groups (15.2 ± 9.8 USH1 and 35.4 ± 24.1 USH2).

The best hearing threshold with their hearing devices was slightly better in USH1 (24.6 dB HL vs. 32.5 dB HL, *p* > 0.05) and we did not observe a significant difference in the results of the speech audiometry in quiet (*p* > 0.05). The difference of the Matrix test SRT between the two groups was also not significant (−3.4 ± 0.5 USH1 vs. −1.7 ± 2.7 USH2, *p* > 0.05).

### 3.3. Localization Test

The mean absolute error was 17.08 ± 15.9° and it was not different between USH1 and USH2 patients. However, we found a significant difference in the time to complete the test (1029 ± 537 s vs. 565.6 ± 224.2 s, *p* < 0.001) and for head movements (89.4 ± 68.7 m vs. 15.4 ± 10.2 m, *p* < 0.001), meaning that USH1 patients were more uncertain in their spatial sound orientation.

### 3.4. Vestibular Examination

The walking age was statistically different between the two groups of children (21.75 ± 3.6 months for USH1 and 13.7 ± 4.7 months for USH2, *p* = 0.008).

At the same time the balance questionnaire score (BQ) was higher in the USH1 group (2.7 ± 1.3 for USH1 vs. 6 ± 4.5 for USH2, *p* = 0.037).

Only one USH1 patient had a positive head-shaking test and a positional horizontal nystagmus at the clinical observation. Concerning VHIT, 3/5 (60%) of USH1 patients and 3/15 (20%) USH2 had a pathologic gain, but the most relevant data was that USH1 had a mean asymmetry percentage of 46 ± 31.5% and USH2 11 ± 8.5% (*p* = 0.001). Consequently, VHIT proved to be a sensitive test for differentiating USH type 1 and type 2 on the base of the vestibular function, as previously demonstrated by other authors [21].

### 3.5. Ophthalmologic Examination

The mean BCVA was 0.89 ± 0.16 for the worse eye and it was not significantly different between the two groups (*p* > 0.05).

The OCT foveal thickness of the worse eye was on average 245 ± 83.6 µm and it was not statistically different between USH1 and USH2 patients (*p* > 0.05). Mean visual field sensitivity was severely reduced in USH1 patients (10.2 ± 6.6 dB) compared to USH2 (23.7 ± 6 dB), *p* = 0.0024. We observed a more severe and early impairment in temporal quadrants, which are the most used in the localization of the lateral stimuli.

### 3.6. Vanderbilt Fatigue Scale

The mean VFS-C was 14.7 ± 9.2 and it was not different in the two groups (*p* > 0.05).

The total score of VFS-P was 15.1 ± 11.1 (7.9 ± 6.4 for mental score, 7.2 ± 5.5 for physical score).

The physical score of VFS-P was statistically different between USH1 (10 ± 5.2) and USH2 (6.4 ± 6.5) patients (*p* = 0.04).

The VSF-M was slightly but significantly related to the age of patients, with higher values for older patients (Pearson’s R = 0.33, *p* = 0.0058). It was not related to the age of diagnosis or the age of first use of hearing aids. The type of hearing device (hearing aids or cochlear implants) did not statistically influence the listening effort measured with the VSF (*p* > 0.05).

It was significantly related to the hearing threshold of the worse ear at the last follow-up visit (R = 0.43, *p* = 0.0045), but not with the best aided hearing curve. Hours counted on the data logging of the hearing device was also an inverse (<hours, >fatigue)-related factor (R = −0.68, *p* = 0.004).

The reported fatigue was not significantly related to the results of the Matrix test. Concerning the localization test, the m-VFS was related to the time of the test (R = 0.52, *p* = 0.0042) and to the performed meters with head movements (R = 0.40, *p* = 0.002), demonstrating that these two parameters of the localization test, which express the child’s uncertainty in locating sounds, go hand in hand with hearing fatigue reported in difficult environments, such as school, by parents and their children.

The Vestibular Function Measured with the VHIT Asymmetry (R = 0.32, *p* = 0.0035) and Balance Problems Reported by Parents (R = 0.51, *p* = 0.005) Were Also Related to the Listening Fatigue.

**Figure 1:**
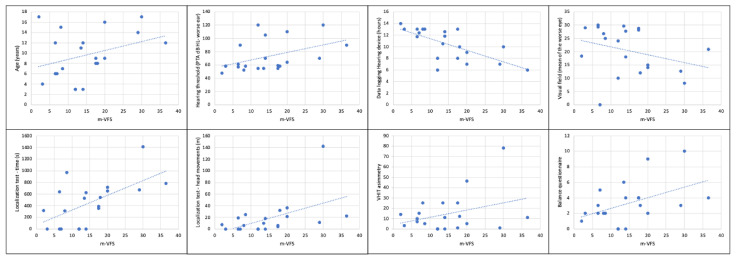
Correlation analysis for mean Vanderbilt fatigue scale questionnaire (m-VFS).

We found a strong inverse relationship between visual field and m-VFS (<retinal sensitivity, >fatigue), probably because in USH patients the impaired visual function, added to the hearing function, has a significant impact on the children’s fatigue in difficult environments (R = 0.78, *p* = 0.0038). Results are depicted in Figure 1.

Analyzing risk factors for listening effort in USH patients (Table 3) we found that USH type 1 had no greater risk of fatigue than USH2. Profound hearing loss, data logging of hearing device < 8 h a day, difficult localization test (head movements > 30 m and time > 650 s), balance problems (balance questionnaire score > 8) and retinal sensitivity < 20 dB, represented risk factors for listening effort measured with VFS. Detailed results are described in Table 3.

## 4. Discussion

Overall, our results about the clinical characteristics of USH patients confirmed previous literature data [5,6,7]. In fact, USH1 patients had profound HL with respect to USH2 patients mostly affected by moderate HL. Nevertheless, two USH2 children were candidates for cochlear implantation because of a rapidly progressive form of hearing loss.

Surprisingly, three USH2 cases had a negative hearing screening result, with a significative diagnostic retardation. In our opinion it was very interesting data because the milder ciliary damage of the hair cell’s structure and function observed in USH2 compared to USH1 animal models [22] could suggest a possible late onset of HL, which would explain the late detection of HL. The hypothesis deserves confirmation on a larger series of cases; future studies may provide insights about the relationships between genotypes and phenotypes, the progression of the disease and the necessary knowledge to detect Usher syndrome early in affected patients.

All patients needed early hearing rehabilitation in childhood, except for one case of USH2 because of mild HL and the opposition from parents.

Despite the profound HL in USH1 patients, all of them had an early diagnosis and rehabilitation start with a cochlear implant, reaching comparable results with respect to USH2 patients regarding speech intelligibility in a noisy environment, as detected with the Matrix test (*p* > 0.05). Consequently, type 1 patients did not show a significantly higher listening effort measured with the Vanderbilt fatigue scale questionnaire, except for the physical fatigue part reported by patients (*p* < 0.05). USH type 1 was not a risk factor of listening effort. Consequently, despite the differences in hearing thresholds between USH1, mostly affected by profound HL, and USH2 children, they could ensure successful outcomes. The results suggested that the early rehabilitation with cochlear implantation in these cases is essential to prevent listening fatigue. Data logging < 8 h a day was also a risk factor for hearing fatigue, underlining the importance of a correct rehabilitation.

At the same time, the significant linear relationship between the subjectively experienced fatigue and the hearing threshold, and visual and vestibular impairment could explain the higher physical fatigue reported by USH1 patients. In fact, USH1 patients demonstrated a higher asymmetry percentage detected with the use of VHIT on the horizontal plane, as previously demonstrated [23], and a worse visual field sensitivity that could affect their spatial awareness and sound localization.

Both visual and balance problems increased the risk of listening effort in our results. It has been demonstrated that chronic unilateral or bilateral vestibular impairment could affect cognitive functions implicated in sound localization especially in difficult environments such as spatial memory, body awareness and the mental representation of the surrounding space [24]. Such cognitive functions become extremely important in visually impaired or blind subjects, who need to use sensory modalities other than vision for spatial localization. As for reflex eye movements, the existence of multisensory integration localized in the superior colliculus of the midbrain is well-documented [25]. At this level, multisensory integration functions by prioritizing the most integral sensory inputs to effectively localize targets [13,14]. This is why listening effort resulted in being inversely related to the visual field sensitivity in the total sample: the progressive restriction of the visual field (“tunnel vision”) that characterizes the syndrome [26] contributes to worsening spatial localization, requiring the use of other sensory resources (such as hearing) and consequently increasing the listening effort.

In our results, USH1 patients had a longer test time localizing sounds on the horizontal plane and higher head movements (*p* < 0.05), which really give an idea of their level of difficulty perceiving sounds in the surrounding space. Both factors were linearly related to the reported listening fatigue in the total sample (Figure 1); the increased test time (>650 s) and movements (>30 m) resulted in being risk factors for the onset of listening fatigue. Consequently, the localization test could be used in the future as an indirect measure of the effort in localizing sounds in the space and added in clinical practice to monitor patients with hearing and vision impairment.

One of the main limits of this study is that the listening effort was measured by the VFS which is a subjective scale. Many other methods could be used, such as adaptive scaling tests for listening effort [27] or behavioral and physiological measures [28]. The variety of methodologies used to measure listening-related fatigue reflects the relative immaturity of the field and the lack of clarity about its clinical meaning which deserves to be explored further with other studies [28]. In the future we hope to evaluate patients using objective methods such as pupil diameter, which has already been tested in our institute [29,30]. Furthermore, this study has been conceived as a retrospective study. We also hope to be able in the future to compare USH data with those of an age-matched control group using a prospective observational case–control model with greater statistical power.

The vestibular assessment was incomplete due to the age of patients, but our intention is to evaluate also the otolith function in patients affected by USH as previous authors demonstrated the pathologic vestibular evoked myogenic potentials in this syndrome [31].

In conclusion, our results demonstrated that listening fatigue in syndromic patients not only referred to the hearing threshold or to the recovery of hearing functions with hearing devices, but also to the integrated hearing, vestibular and visual functions which together contribute to the complex cognitive network that guides sound localization in the space and in difficult situations. The quantification in each patient of the exact contribution to the listening effort of each sensory modality (auditory, vestibular, visual and localization), and the degree of impairment of each of these pathways, is fundamental if we consider the theory of “sensory-substitution” to try to maximize the most conserved sense or exploit other sensory modalities (vibro-tactile). This suggests the necessity for multimodal rehabilitation and multidisciplinary care in Usher syndrome patients. In this kind of educational approach, the use of the most integral sensorial systems needs to be encouraged through proper stimulation programs. In particular, those among the auditory, vestibular and visual systems that are most likely to remain stable with aging. Results should also be considered to support hearing impaired children, especially in cases of multiple comorbidities, to improve school environments and teachers’ awareness, and promote learning.

## Figures and Tables

**Table 1:** Demographic characteristics and details of the DNA variants identified in USH patients, sub-grouped in USH1 (a) and USH2 (b). Mat, maternal; pat, paternal; Y, yes; N, no; *, sibs: 3–4, 10–11 and 13–14 are three couples of sibs; P, DNA variant identified through gene panel NGS sequencing and segregation analysis. Other variants were detected by whole exome sequencing (WES) of the proband and parents (trio WES); P, pathogenic; LP, likely pathogenic; VUS, variant of uncertain significance.

a. USH1				Allele 1						Allele 2				
Patient ID	Sex	Age (yrs)	Gene	Reference Sequence	cDNA Position	AA Change	Inheritance	ACMG Classification	Reported	cDNA Position	AA Change	Inheritance	ACMG Classification	Reported
2	m	6	CDH23	NM_022124.6	c.9433C>T	p.(Gln3145*)	pat	P (5)	Y	c.5712G>A	p.(Thr1904Thr)	mat	P (5)	Y
7	f	16	MYO7A	NM_000260.4	c.3719G>A	p.(Arg1240Gln)	pat	P (5)	Y	c.6028G>A	p.(Asp2010Asn)	mat	P (5)	Y
9	f	3	CDH23	NM_022124.6	c.3646_3647delCT	p.(Leu1216Glyfs*41)	pat	LP (4)	N	c.4562A>G	p.(Asn1521Ser)	mat	LP (4)/P (5) in ClinVar/P (5) in DVD, but associated with NSHL; VUS (3) in HGMD	Y
17	f	3	USH1C	NM_005709.4	c.711delT	p.(Phe237Leufs*5)	mat	LP (4)	N	c.711delT	p.(Phe237Leufs*5)	pat	LP (4)	N
18	m	17	CDH23	NM_022124.6	c.5985C>A	p.(Tyr1995*)	mat	P (5)	Y	c.5985C>A	p.(Tyr1995*)	pat	P (5)	Y
b. USH2				Allele 1						Allele 2				
1	m	12	ADGRV1	NM_032119.4	c.13655dupT	p.(Asn4553Glufs*18)	mat	P (5)/DM? in HGMD	Y	c.9447+1G>A	p.?	pat	LP (4)	N
3 *	m	14	USH2A	NM_206933.4	c.11864G>A	p.(Trp3955*)	pat	P (5)	Y	c.6705_6708del	p.(Asp2237Argfs*41)	mat	P (5)	Y
4 *	m	12	USH2A	NM_206933.4	c.11864G>A	p.(Trp3955*)	pat	P (5)	Y	c.6705_6708del	p.(Asp2237Argfs*41)	mat	P (5)	Y
5	f	7	USH2A	NM_206933.4	c.2276G>T	p.(Cys759Phe)	pat	P (5)	Y	c.11864G>A	p.(Trp3955*)	mat	P (5)	Y
6	f	6	USH2A	NM_206933.4	del of exons 5-->10		mat	P (5)	Y	del of exons 5-- >10		pat	P (5)	Y
8	f	8	ADGRV1	NM_032119.4	c.10084C>T	p.(Gln3362*)	pat	P (5)	Y	c.13655dup	p.(Asn4553Glufs*18)	mat	P (5)	Y
10 *	m	17	USH2A^P^	NM_206933.4	c.11864G>A	p.(Trp3955*)	mat	P (5)	Y	c.11864G>A	p.(Trp3955*)	pat	P (5)	Y
11 *	m	15	USH2A^P^	NM_206933.4	c.11864G>A	p.(Trp3955*)	mat	P (5)	Y	c.11864G>A	p.(Trp3955*)	pat	P (5)	Y
12	f	4	USH2A	NM_206933.4	c.232T>G	p.(Phe78Val)	pat	LP (4)/VUS (3)	Y	c.13392G>A	p.(Trp4464*)	mat	P (5)/LP (4)	Y
13 *	f	3	USH2A	NM_206933.4	c.5199delATATGTTTC AT	p.(Tyr1730Trpfs*6)	pat	P (5)	Y	c.9270C>A	p.(Cys3090*)	mat	P (5)	Y
14 *	m	9	USH2A	NM_206933.4	c.5199delATATGTTTC AT	p.(Tyr1730Trpfs*6)	pat	P (5)	Y	c.9270C>A	p.(Cys3090*)	mat	P (5)	Y
15	f	11	USH2A	NM_206933.4	c.9270C>A	p.(Cys3090*)	pat	P (5)	Y	c.1876C>T	p.(Arg626*)	mat	P (5)	Y
16	m	9	USH2A	NM_206933.4	c.2099_2120delGGA CAGTGGATGGAGATA TTAC	p.(Gly700Alafs*49)	pat	LP (4)	N	c.8167C>T	p.(Arg2723*)	mat	P (5)	Y
19	f	12	ADGRV1	NM_032119.4	c.13655dupT	p.(Asn4553Glufs*18)	pat	P (5)	Y	c.4378G>A	p.(Gly1460Ser)	mat	P (5)	Y
20	m	8	USH2A^P^	NM_206933.4	c.1055C>T	p.(Thr352IIe)	mat	P (5)	Y	c.1055C>T	p.(Thr352IIe)	pat	P (5)	Y

**Table 2:** Clinical characteristics in USH patients. yrs, years; HL, hearing loss; HA, hearing aids; m, meters; s, seconds; VHIT, video head impulse test.

Patient ID	Age (yrs)	Sex	Diagnosis	Age HL Diagnosis (Months)	PTA—Worst Ear (dB HL)	First HA Use (Months)	Data Logging of Hearing Device	Localization Test—Head Movements (m)	Localization Test—Time (s)	VHIT Asymmetry (%)	Balance Questionnaire	Visual Field (dB)
N1	12	M	USH2C	30	57	36	13	19	640	7	3	29.35
N2	6	M	USH1	0	90	20	12.4	/	/	15	5	0.00
N3	14	M	USH2A	24	70	28	7	11.7	672.55	1	3	12.65
N4	12	M	USH2A	0	90	60	6	22.52	782.15	11	4	20.87
N5	7	F	USH2A	0	58	51	13	25	968.75	5	2	25.00
N6	6	F	USH2A	0	61	53	11.7	/	/	10	2	30.00
N7	16	F	USH1B	0	110	19	9	36.7	650	46	9	14.99
N8	8	F	USH2C	0	58	8	10	32	543	12	3	12.00
N9	3	F	USH1D	0	120	5	8	/	/	/	/	10.00
N10	17	M	USH2A	60	47.5	60	14	7.73	313.95	14	1	18.34
N11	15	M	USH2A	0	52.5	12	13	6.28	309.95	25	2	26.82
N12	4	F	USH2A	0	58	/	13	/	/	3	2	29.00
N13	3	F	USH2A	0	55	4	6	/	/	/	/	24.00
N14	9	M	USH2A	0	55	6	8	6.21	355.15	1	4	28.12
N15	11	F	USH2A	0	55	31	10.5	10.07	527.35	25	6	29.67
N16	9	M	USH2A	54	64	69	7	21.78	715.55	5	2	14.00
N17	3	F	USH1C	0	105	5	12.6	/	/	/	/	18.00
N18	17	M	USH1D	0	120	27	10	142.26	1409.55	78	10	8.11
N19	12	F	USH2C	48	70	66	11.8	18.28	622.95	11	4	27.68
N20	8	M	USH2A	12	59	12	13	4.15	387.95	25	4	28.82

**Table 3:** Risk factors for listening effort in USH patients. VFS-C, Vanderbilt fatigue scale children; VFS-P, Vanderbilt fatigue scale parents; OR, odd ratio; CI, confidence interval; *, *p* < 0.05.

		USH1	Hearing Loss > 90 db HL	Data Logging < 8 h/day	Localization Test (Head Movements > 30 m)	Localization Test (Time > 650 s)	VHIT Asymmetry > 20	Balance Questionnaire ≥ 8	Retinal Sensitivity < 20 dB
VFS-C ≥ 27	% exposed cases	50%	75%	75%	50%	100%	50%	50%	75%
	% exposed controls	18%	18%	12%	6%	25%	12%	6%	31%
	OR	1.66	13	21	15	120	7	15	6.6
	*p* value	0.19	0.028 *	0.009 *	0.028 *	0.007 *	0.09	0.028 *	0.11
	CI	0.4–44.4	0.1–172.9	1.4–314	0.9–251	0.2–70,528	0.6–81.7	0.9–251	0.5–80
VFS-P Physical ≥ 13	% exposed cases	50%	75%	75%	50%	100%	50%	50%	75%
	% exposed controls	18%	18%	12%	6%	25%	12%	6%	18%
	OR	1.66	13	21	15	120	7	15	13
	*p* value	0.19	0.028 *	0.009 *	0.028 *	0.007 *	0.09	0.028 *	0.028 *
	CI	0.4–44.4	0.1–172.9	1.4–314	0.9–251	0.2–70,528	0.6–81.7	0.9–251	0.1–172.9
VFS-P Mental ≥ 15	% exposed cases	50%	100%	50%	50%	100%	50%	50%	50%
	% exposed controls	25%	11%	11%	11%	11%	22%	5%	33%
	OR	3	160	8	8	160	3.5	17	2
	*p* value	0.32	0.003 *	0.13	0.13	0.003 *	0.37	0.04 *	0.63
	CI	0.31–28.8	1.2–108,466	0.3–184.4	0.3–184.4	1.2–108,466	0.2–69.3	0.5–523.8	0.11–37.8

## Data Availability

The data presented in this study are available on request from the corresponding author.
